# Ziehl-Neelsen Staining Technique Can Diagnose Paragonimiasis

**DOI:** 10.1371/journal.pntd.0001048

**Published:** 2011-05-17

**Authors:** Günther Slesak, Saythong Inthalad, Phadsana Basy, Dalaphone Keomanivong, Ounheaun Phoutsavath, Somchaivang Khampoui, Aude Grosrenaud, Vincent Amstutz, Hubert Barennes, Yves Buisson, Peter Odermatt

**Affiliations:** 1 Service Fraternel d'Entraide, Vientiane, Lao People's Democratic Republic (Lao PDR); 2 Tropenklinik Paul-Lechler-Krankenhaus, Tübingen, Germany; 3 Luang Namtha Provincial Hospital, Luang Namtha, Lao People's Democratic Republic (Lao PDR); 4 Institut de la Francophonie pour la Médecine Tropicale, Vientiane, Lao People's Democratic Republic (Lao PDR); 5 Swiss Tropical and Public Health Institute, Basel, Switzerland; 6 University of Basel, Basel, Switzerland; Khon Kaen University, Thailand

## Abstract

**Background:**

We evaluated the Ziehl-Neelsen staining (ZNS) technique for the diagnosis of paragonimiasis in Laos and compared different modifications of the ZNS techniques.

**Methodology:**

We applied the following approach: We (1) examined a paragonimiasis index case's sputum with wet film direct examination (WF) and ZNS; (2) re-examined stored ZNS slides from two provinces; (3) compared prospectively WF, ZNS, and formalin-ether concentration technique (FECT) for sputum examination of patients with chronic cough; and (4) compared different ZNS procedures. Finally, we assessed excess direct costs associated with the use of different diagnostic techniques.

**Principal Findings:**

*Paragonimus* eggs were clearly visible in WF and ZNS sputum samples of the index case. They appeared brownish-reddish in ZNS and were detected in 6 of 263 archived ZNS slides corresponding to 5 patients. One hundred sputum samples from 43 patients were examined with three techniques, which revealed that 6 patients had paragonimiasis (13 positive samples). Sensitivity per slide of the FECT, ZNS and the WF technique was 84.6 (p = 0.48), 76.9 (p = 0.25) and 61.5% (p = 0.07), respectively. Percentage of fragmented eggs was below 19% and did not differ between techniques (p = 0.13). Additional operational costs per slide were 0 (ZNS), 0.10 US$ (WF), and 0.79 US$ (FECT). ZNS heated for five minutes contained less eggs than briefly heated slides (29 eggs per slide [eps] vs. 42 eps, p = 0.01). Bloodstained sputum portions contained more eggs than unstained parts (3.3 eps vs. 0.7 eps, p = 0.016).

**Conclusions/Significance:**

*Paragonimus* eggs can easily be detected in today's widely used ZNS of sputum slides. The ZNS technique appears superior to the standard WF sputum examination for paragonimiasis and eliminates the risk of tuberculosis transmission. Our findings suggest that ZNS sputum slides should also be examined routinely for *Paragonimus* eggs. ZNS technique has potential in epidemiological research on paragonimiasis.

## Author Summary

Lung fluke (*Paragonimus*) infection causes similar symptoms to pulmonary TB and is an important differential diagnosis in endemic areas. Standard diagnosis is wet film (WF) microscopic examination of sputum samples. For the last fifty years, Ziehl-Neelsen stain (ZNS) has been believed to destroy *Paragonimus* eggs. However, our investigation of stored ZNS slides and our prospective comparison of wet film, ZNS, and formalin-ether concentration technique of sputum of chronic cough patients in Laos showed that (1) similarly to wet film and FECT, *Paragonimus* eggs were hardly fragmented by ZNS; and (2) ZNS had a higher nominal sensitivity for detection of *Paragonimus* eggs than WF at lowest costs. Examination of bloody sputum parts revealed more eggs; while on the other hand, ZNS with continuous heating of the slides reduced the quantity of eggs compared to the current heating technique. Further, ZNS should also be investigated with the 10× lens for *Paragonimus* eggs, in addition to the 100× lens for TB, to reduce misdiagnosis of sputum-negative TB. Finally, the ZNS methodology appears to diminish biosafety risks of the standard wet film procedure. ZNS could be a valuable technique in epidemiological research on paragonimiasis.

## Introduction

Paragonimiasis is a primary pulmonary food-borne trematodiasis and zoonosis present in numerous countries, especially in tropical Asia where 293 million people are estimated at risk of infection [1]. Causing symptoms similar to pulmonary tuberculosis (TB), it is frequently misdiagnosed and treated as sputum-negative TB [2]–[5]. Standard diagnosis in endemic areas relies on sputum examination by direct microscopy of fresh sputum (wet film mount, WF) and concentration techniques such as formalin-ether concentration technique (FECT), as well as stool sample examinations [3], [6]–[9]. In 1960, Sadun and Buck reported from their studies in South Korea that only debris of *Paragonimus* eggs were found in Ziehl-Neelsen stained (ZNS) sputum slides [7]. Since then, *Paragonimus* eggs diagnosis based on ZNS sputum has been abandoned [4], [5], [9]. In the meantime, however, there have been numerous modifications of the ZNS technique [10], especially the use of different decolorizers such as sulphuric acid [11] and hydrochloric acid-alcohol [9], [12]. Furthermore, different durations of heat application during the carbol-fuchsin staining process have been introduced and investigated, ranging from a single period of a few seconds - as in current practice [9], [13] - to continuous heating of the slide for several minutes (e.g. 5 minutes as described in 1976) [12]. However, it is unknown which ZNS modification was used by Sadun and Buck [7]. Additionally, the WF technique has the potential for TB transmission, and thus poses an obvious biosafety hazard. Furthermore, a reliable later quality control of the WF cannot be performed after the slide has dried up. In practice it is often only considered after a negative TB examination at a time when the sputum sample usually is discarded.

Lao People's Democratic Republic (Laos, Lao PDR) is endemic for paragonimiasis and TB [3], [4], [14]–[17]. In May 2009, a local farmer was examined at the Luang Namtha (LN) provincial hospital, Northern Laos, with a four-year history of cough and haemoptysis. Microscopic analysis revealed an extraordinary high number of *Paragonimus* eggs in the direct sputum examination, also clearly detected in the ZNS slides. It was this surprising confirmation of the wet film analysis by the ZNS slides that prompted our interest in re-evaluating the current sensitivity of ZNS technique for the diagnosis of paragonimiasis.

The objective of our study was to evaluate the ZNS procedure as a diagnostic tool for paragonimiasis in sputum samples in comparison to different currently used diagnostic techniques, namely the WF and FECT, and to compare different historic and current modifications of the ZNS technique for the detection of *Paragonimus* eggs.

## Methods

### Examination of paragonimiasis index case

In August 2009, a paragonimiasis index case was diagnosed by WF sputum examination in Luang Namtha provincial hospital, Northern Laos. Two sputum samples were examined with four different diagnostic techniques: (i) the standard WF (2 WF slides, 1 slide per sputum) employing a magnification of 40× and 100× [9]; (ii) the ZNS [11], [13] (2 ZNS slides, 1 slide per sputum), where samples were examined using a magnification of 40×, 100×, and 1000×; (iii) the auramine staining (2 AS slides, 1 slide per sputum) using fluorescence microscopy with a magnification of 600× [18]; (iv) the examination of an additional sputum sample with and without the bleach concentration technique, a newer method which has lately been suggested to improve the TB detection rate in Laos [13].

### Examination of archived Ziehl-Neelsen stained slides

We re-examined ZNS slides for the presence of *Paragonimus* eggs from suspected TB patients. These slides were stored in the laboratories of the provincial tuberculosis program of LN province, Northern Laos, and Attapeu province, Southern Laos. The analyses were carried out by one trained laboratory technician/doctor using a magnification of 100×. Positive slides were double-checked by a second laboratory technician and photo-documented.

### Validity of Ziehl-Neelsen staining technique to detect *Paragonimus* eggs

We collected sputum samples taken on two consecutive days from patients with chronic cough (>two weeks) in LN province, from September 2009 until April 2010, according to the Lao TB guidelines [11]. Included were patients from the index case's village (Phonthong), and from other villages where previously paragonimiasis patients were detected or suspected. Furthermore, we enrolled chronic cough patients from LN provincial hospital, and Vieng Phoukha and Muang Sing district hospitals.

One slide from each sputum sample was examined using WF, ZNS and FECT. Two independent laboratory technicians at the LN provincial hospital examined each slide in a blinded way. The technicians were not aware of the identity of the patient and the results of previous examinations (coded slides). In addition, they were working in separate rooms without the possibility to communicate. The slides were given random numbers and kept in a closed box with no further indications while being provided one by one to the technicians. The number of *Paragonimus* eggs detected per slide was recorded in separate booklets. One of us (GS) ensured that blinding procedures were respected. After unblinding discordant slides were rechecked, results confirmed by a third laboratory technician, and detected eggs photo-documented.

**Figure 1:**
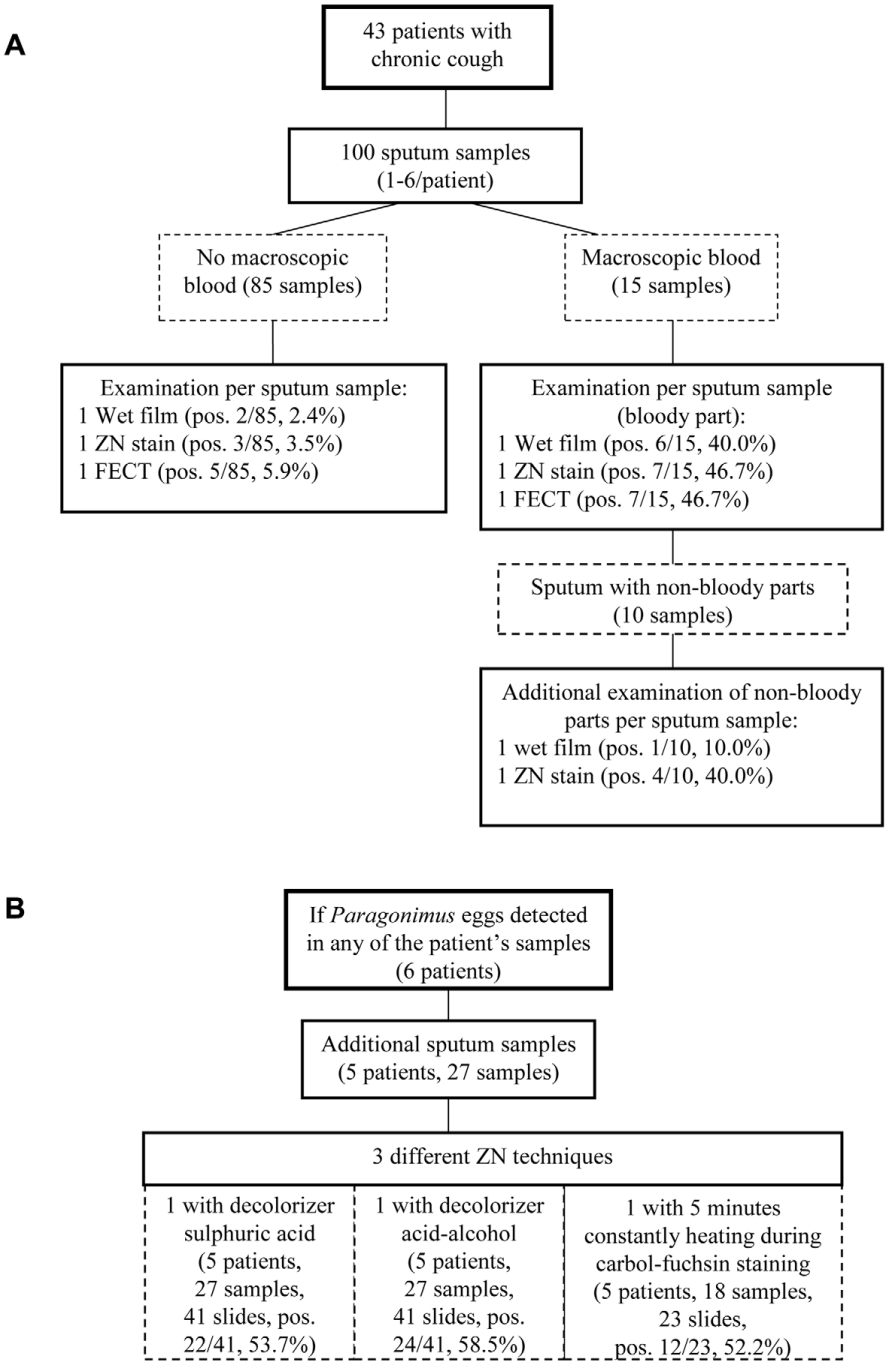
Study flow charts of the prospective investigation of the Ziehl-Neelsen technique. Study flow chart of the evaluation of the Ziehl-Neelsen technique to diagnose *Paragonimus* spp. eggs in Luang Namtha, Laos, using sputum samples of patients with chronic cough (A). Comparison of different Ziehl-Neelsen staining procedures in egg positive samples (B).

From blood stained sputum samples with clearly defined non-bloody parts two sets of WF and ZNS slides were established; one from the bloody and one from the non-bloody sputum portion (Figure 1A).

More sputum samples were asked from *Paragonimus* eggs-positive patients and as many sets as possible performed of: 1 wet film, 1 ZNS using sulphuric acid as decolorizer [11], [13], and 1 ZNS using hydrochloric acid-ethanol as decolorizer [9]. In addition, a subsample of sputum was processed using the historical ZNS procedures with continuous heating during the carbol-fuchsin staining process [12] (Figure 1B).

### Ethics statement

The study was approved by the National Ethics Committee for Health Research, Ministry of Health, Vientiane, Laos (No. 272/NECHR). All patients were counseled and provided written informed consent prior to enrollment. In case of detection of AFB or *Paragonimus* eggs, the patient was explained the findings and treated according to the Lao TB guidelines [9], [11]. Sputum negative patients were referred to the provincial hospital for further diagnosis and treatment. All paragonimiasis patients were treated with praziquantel (75 mg/kg/day for 3 days) according to international standards [4], [14], [16]. TB patients were treated according to the guideline of the National TB Control Program [11].

### Laboratory procedures

Laboratory procedures were performed according to standards. For direct examination, sputum was transferred on microscopic slides, covered with a cover slide, and examined with a magnification of 100× (10× objective) [9]. The standard ZNS “hot staining” was performed according to the Lao TB guidelines [11] with sulphuric acid as decolorizer and only briefly heated (until it started to steam) at the beginning of the carbol-fuchsin staining [13]. Other ZNS slides were continuously heated and kept steaming during the total five minutes of the carbol-fuchsin staining process [12]. Another ZNS technique used (instead of sulphuric acid) acid alcohol to decolorize slides [9]. All ZNS slides were examined with a magnification of 100× (10× objective) for *Paragonimus* eggs and with a magnification of 1000× (100× objective with oil) to identify acid-fast bacilli (AFB). For the FECT, sputum was homogenized with 0.9% NaCl, 10% formalin added, mixed and centrifuged. Supernatant was discarded, 0.9% NaCl and ether added, mixed, centrifuged, and the sediment examined as for direct examination [9]. Auramine staining and the bleach method were performed as described by Trusov *et al*. [18] and Ongkhammy *et al*. [13], respectively. The number of normal and fragmented *Paragonimus* eggs were identified and recorded per slide.

### Cost analysis

Calculation of average costs per slide included operating costs (working time, chemicals, disposable materials, electricity) but not capital costs (laboratory equipment such as centrifuge, vortex mixer for the FECT) presuming its availability in a laboratory offering ZNS. Working time was estimated based on the used standard operating procedures; time for microscopy was assumed as equal for all techniques and depending on the examiner's experience and therefore not included. Yearly costs were calculated for the number of about 1000 sputum samples examined for TB at LN provincial hospital taking into account time savings for grouped sample testing.

### Data management and analysis

Data were entered in EpiData (version 3.1, the EpiData Association, Odense, Denmark). All records were cross-checked against original data sheets. Statistical analysis was performed with GraphPad Instat and QuickCalcs (GraphPad Software, California, USA). Agreements between the two readings were assessed with Cohen's Kappa (κ) coefficient. Paired categorical variables were compared using McNemar's test. Wilcoxon ranksum test and Friedman test were performed for comparison of two and three continuous variables, respectively. 95% confidence intervals (95% CI) were calculated for continuous and categorical data. The diagnostic “gold standard” for a *Paragonimus* spp. infection was defined as detection of at least one *Paragonimus* spp. egg in any of the examinations (three techniques) per sputum sample. Sensitivity and negative predictive value (NPV), and inter-observer's agreement of one slide's examination for the detection of *Paragonimus* eggs was calculated for each diagnostic technique (WF, ZNS, and FECT).

## Results

### Observations on sputum of index case

**Figure 2:**
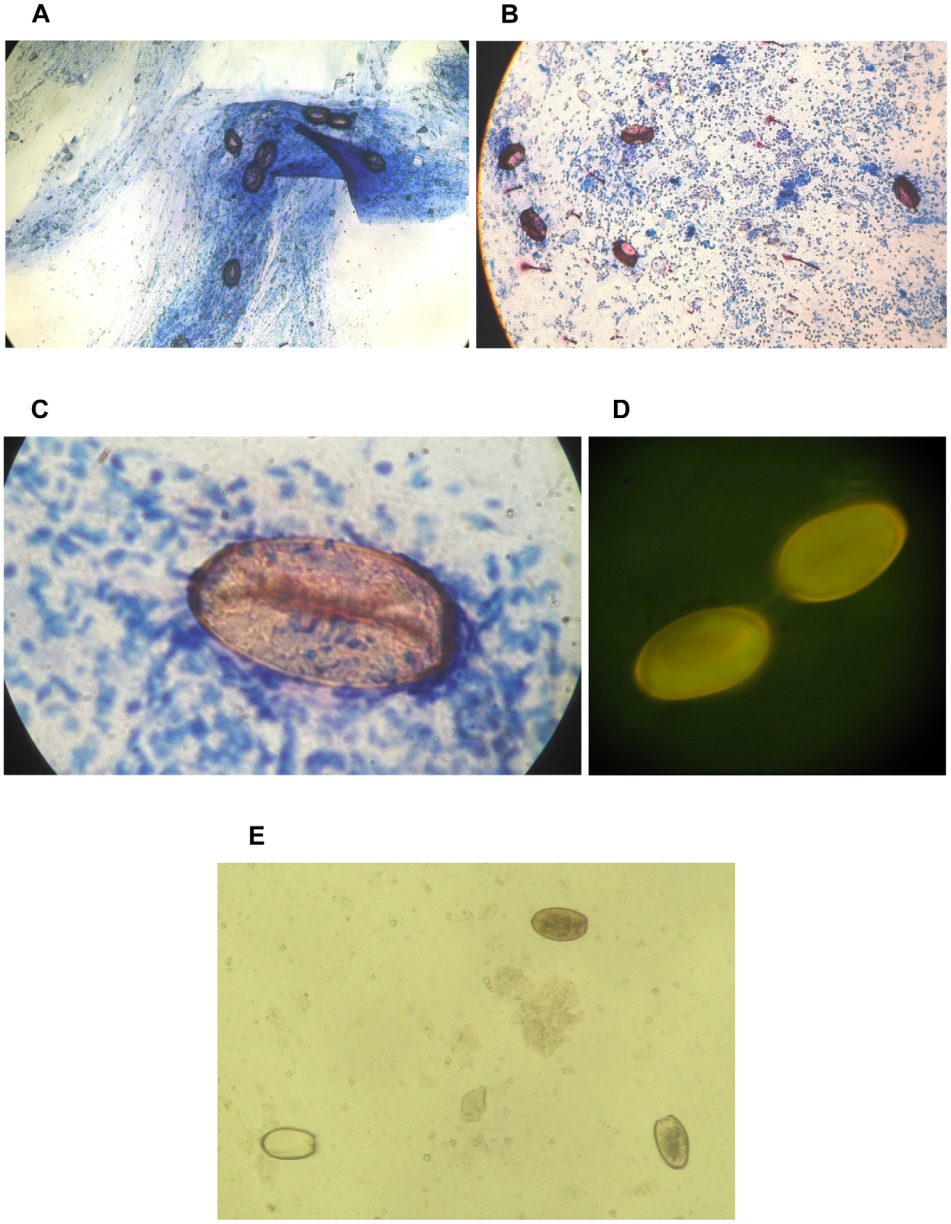
Microscopic appearance of *Paragonimus* eggs after different staining techniques. Brownish to reddish colored *Paragonimus* eggs in Ziehl-Neelsen stained sputum (10× objective, 100× magnification) (**A** and **B**). *Paragonimus* egg in the Ziehl-Neelsen stained sputum with 100× objective (1000× magnification) (**C**). Auramine stain examined by fluorescence microscopy with 60× objective (600× magnification) (**D**). Wet slide after the bleach concentration technique (**E**); from left to right: *Paragonimus* egg that is empty, one that appears unchanged, and one egg without the operculum examined with 10× objective (100× magnification).

In the ZNS sputum slides, *Paragonimus* eggs appeared in a brownish to reddish color with often one or two convex or concave inner lines resembling a deflated American football. Specific characteristics of the *Paragonimus* spp. eggs were clearly visible such as the operculum and shoulders, the thick walls and the three dimensional shape (Figure 2A–C). The auramine stained slide showed much fewer but similar eggs (7 and 6 *versus* ZNS 47 and 67, and WF 146 and 162 eggs, Figure 2D). *Paragonimus* specific features were clearly visible. The bleach concentration technique mostly altered *Paragonimus* eggs in the direct microscopy (Figure 2E). However, all eggs remained clearly identifiable and 25 of 117 observed eggs (21.4%) were unchanged. The remaining eggs were either empty, fragmented or had open opercula.

When the bleach concentration technique was combined with the ZNS or the auramine stain the slide that was further stained by the ZNS revealed only 4 eggs. When further stained by auramine stain not a single egg was detected.

### Examination of archived ZNS slides

In June and July 2009, we examined 211 ZNS slides produced between January and March 2009 for the presence of *Paragonimus* eggs. The patients all originated from five districts of the Attapeu province. We identified *Paragonimus* eggs in four of 211 slides (1.9%). The slides belonged to four different patients in whom the diagnosis of paragonimiasis had not been done before.

In February 2010, we examined, 52 ZNS slides produced between October and December 2009 at the Muang Sing district hospital, LN province. In two of these slides (3.8%) we found *Paragonimus* eggs. Both slides belonged to an already diagnosed paragonimiasis patient.

### Validity of Ziehl-Neelsen staining technique to detect *Paragonimus* eggs and diagnose paragonimiasis

We identified 43 patients with chronic cough which we enrolled in the study (Figure 1A). In total, one hundred sputum samples were obtained (mean 2.3 samples per patient; range: 1–6). Fifteen sputum samples contained macroscopic blood. In ten of these samples bloody parts were clearly distinguishable from non-bloody parts of which extra sets of WF and ZNS slides were performed. Thirteen of one hundred samples of six patients had *Paragonimus* eggs in at least one of the slides. One additional patient with paragonimiasis was only diagnosed in a sample examined outside of the study but not in the 2 included samples.

Patients' age, sex, and symptoms did not differ except previous consumption of raw or insufficiently cooked crabs (3 of 7 *Paragonimus* positive patients, 42.9%, vs. 3 of 36 *Paragonimus* negative patients, 8.3%, p = 0.045, Table 1).

**Table 1:** Characteristics of study participants (n = 43) from Luang Namtha province, Laos.

	*Paragonimus* egg positive (n = 7)^a^N (%)	*Paragonimus* egg negative (n = 36)N (%)
Female	4 (57.1)	15 (41.7)
Age in years (95%CI)	32.6 (11.7–53.4)	41.2 (36.1–46.5)
Number of Villages	7	11
Number of Districts	3	4
AFB positive sputum	1 (14.3)	2 (5.6)
Reported History:		
Duration of cough in months (95%CI)	29.1 (7.4–50.9)	19.5 (12.3–26.7)
Blood in sputum (yes)	6 (85.7)	22 (61.1)
Brownish sputum (yes)	3 (42.9)	16 (44.4)
Fever episodes (yes)	0	13 (36.1)
Night sweats (yes)	2 (28.6)	11 (30.6)
Loss of weight (yes)	3 (42.9)	22 (61.1)
Loss of appetite (yes)	4 (57.1)	17 (47.2)
Consumption of raw or insufficiently cooked crabs (yes) b	3 (42.9)	3 (8.3)

a : Including 1 patient whose 2 sputum samples were negative but had *Paragonimus* eggs in a sample examined outside of the study.

b : p = 0.045, all other variables p>0.05.

AFB: Acid-fast bacilli.

The results on the validity of the different diagnostic techniques to identify *Paragonimus* eggs are shown in Table 2. Sensitivity was lowest in the WF and highest in the FECT.

**Table 2:** Statistical comparison of the different diagnostic techniques.

	Sensitivity% (n/n)	NPV% (n/n)	P-value (McNemar's test)	Kappa statistics(95%CI)
Formalin-Ether Concentration (n = 100)	84.6 (11/13)	97.8 (87/89)	0.48	0.89 (0.74–1.04)
Ziehl-Neelsen (n = 100)	76.9 (10/13)	96.7 (87/90)	0.25	0.73 (0.47–0.99)
Wet film(n = 100)	61.5 (8/13)	94.6 (87/92)	0.074	1.00

NPV: negative predictive value.

CI: confidence interval.

Sensitivity and negative predictive values (NPV) of one slide examination for the detection of *Paragonimus* eggs in sputum samples (n = 100) of patients with chronic cough.

The mean number of *Paragonimus* eggs per slides (eps) in WF (2.23 eps), ZNS (1.95 eps) and FECT (4.95 eps) were not statistically different (p = 0.34). The mean rate of at least partly fragmented eggs varied considerably from 18.3% (range 0–50%) in WF, 10.7% (0–100%) in FECT, and 12.5% (0–100%) in ZNS but showed no significant difference (p = 0.13).

Average operational costs per slide were calculated for consumables at 0.09 US$ and 0.65 US$ for WF and FECT, respectively. Additional costs for working time was 0.01 US$ (2 minutes), and 0.14 US$ (21 minutes) for WF and FECT respectively. No additional costs occur in ZNS staining procedures. Overall, yearly additional costs of 100 US$ (25 hours) and 692 US$ (200 hours) occur for WF and FECT, respectively.

Fifteen sputum samples contained macroscopic blood, of which 10 had both bloody and non-bloody parts (Figure 1A). Eight of these ten samples belonged to patients diagnosed with paragonimiasis. Slides performed from bloody parts of paragonimiasis patients' samples (8 WF, 8 ZNS) showed a higher mean number of eggs than from areas without blood (3.3 eps, range 0–10 eps, 95%CI 1.3–5.4 *versus* 0.7 eps, range 0–5 eps, 95%CI 0–1.4, p = 0.016).

Comparison of the different ZNS techniques by additional sputum samples (Figure 1B, n = 27) revealed more eggs per slides in standard ZNS (41.9 eps, 95% CI 5.5–78.3) compared to the technique using continuous heating during the carbol-fuchsin staining process (29.3 eps, 95% CI 4.1–54.4, each n = 23, p = 0.01). The number of eggs per slide detected with the two different decolorizers was not statistically different (sulphuric-acid: 24.7 eps, 95% CI 4.0–45.4 versus acid-alcohol: 29.7 eps, 95% CI 2.1–57.3, each n = 41, p = 0.51). The rate of fragmented eggs did not differ between the different ZNS techniques (standard ZNS 1.3% (13 of 964 eggs) *versus* ZNS with continuous heating 1.9% (13 of 673 eggs, n = 23, p = 0.44); standard ZNS 1.5% (15 of 1011 eggs) *versus* ZNS with acid-alcohol decolorizer 1.9% (23 of 1217 eggs, n = 41, p = 0.52)).

## Discussion

Our study showed that the currently widely used ZNS technique for AFB diagnosis is able to detect *Paragonimus* eggs. Furthermore, we provide evidence that its sensitivity might even be higher than the WF technique which is today's parasitological reference technique for paragonimiasis and we found that FECT appears superior to WF for paragonimiasis diagnosis. However, the costs related to latter technique highlights the disadvantage of FECT as special technical material and additional time are required. In addition to validity and costs, safety concerns must be considered. WF working procedures exposes laboratory staff to potentially infectious agents, i.e. AFB. FECT includes the utilization of ether which is an additional, non-neglectable hazard in a laboratory that uses open fire for ZNS technique. FECT is therefore not available as a routine diagnostic test in health services in Laos and we would only recommend it as a test for paragonimiasis in specialized settings where this technique already has been well established, e.g. central referral laboratories.

Sadun and colleagues 1960 [7] described that in 20% of the microscopically diagnosed cases with pulmonary paragonimiasis eggs were found only after numerous direct examinations; in one case only after the 27^th^ examined sample. Repetition of direct parasitological tests has successfully been used for diagnosis of other trematode infections; an examination of a second and third slide had increased *Schistosoma mansoni* egg positivity from 64.8 to 74.3 and 83.8% [19]. This indicates that examination of further sputum samples with the ZNS technique, which, according to the national TB guidelines, would anyways need to be done, might be much more cost-effective and more appropriate than to invest in a more sophisticated method like the FECT with a possibly slightly higher detection rate.

Currently, the simple and cheap WF microscopy is still the standard examination for paragonimiasis in most developing countries including Laos [3], [4], [9], [14]–[16]. However, it has several disadvantages compared to the ZNS technique: first, processing potentially infective material can further increase the already existing higher risk of TB transmission among laboratory workers in low-income countries lacking appropriate control measures [20]. Second, quality control by another laboratory technician is difficult because slides quickly dry up and cannot be stored and re-read. Finally, in the routine work at health services paragonimiasis is only considered when TB examination is negative. At this time point the sputum sample is already discarded, and further slides for diagnosis can not be established any more.

In contrast, ZNS slides are recorded and stored for external quality control according to the TB policy and can be reviewed later, without safety concerns. The successful diagnosis of several patients by reexamination of archived ZNS slides demonstrates that *Paragonimus* eggs are preserved on the ZNS slides. At this stage we do not know for which time period these eggs remain visible. However, each egg identified and paragonimiasis case detected provides information on an existing focus of transmission and a community based follow-up can be launched. This method has the potential to be applied in epidemiological research on paragonimiasis, e.g. estimations of infection prevalence, identification of endemic areas and more. Serological examinations show a higher sensitivity but are usually not available in developing countries. Furthermore, they are prone to overestimate infection rates due to possible persistent antibodies and cross-reactions with other helminthic infections [21]. A definitive diagnosis of paragonimiasis is still carried out by the demonstration of lung-fluke eggs in sputum, feces, or thoracic tissue [21], [22].

The mucus in the ZNS and the auramine staining of sputum helps to keep the eggs attached to the slide. During the bleach concentration technique, mucus and fibers are resolved [13]. This may explain why slides processed with ZNS after bleaching yielded only very few *Paragonimus* eggs.

We recovered higher numbers of eggs from bloody compared to non-bloody parts of the sputum specimen. This proves to be a simple way of improving the pretest likelihood as it is suggested in general text books [23]. *Paragonimus* infected lungs contain nodular areas with necrosis and numerous eggs. Adjacent richly perfused granulation tissue is the basis for hemorrhagic pneumonia [6], [24]. In potentially paragonimiasis endemic areas direct examination should preferably be done from bloody portions of sputum.

One patient had a co-infection of TB and *Paragonimus* which highlights the importance of correct diagnostic procedures for both diseases. An integration of routine ZNS examinations for *Paragonimus* eggs could help to avoid misdiagnosis of sputum-negative TB due to Paragonimiasis [2]–[5] in endemic areas and contribute to correct diagnosis of co-infections. As for TB, paragonimiasis diagnosis requires repeated sputum examinations [7]. As such, the ZNS technique represents the ideal common diagnostic procedure.

However, why did Sadun and Buck [7] find only debris of *Paragonimus* eggs in the ZNS? The ZNS techniques have evolved over the last decades [9], [10], [12] which might be one of the reasons that nowadays *Paragonimus* eggs can indeed be found. We detected a significantly lower number of eggs in those slides that were continuously heated during the carbol-fuchsin staining process. Evidently, extensive heat can degenerate the egg wall proteins. The type of decolorizer did not influence the detection of eggs. There might be other factors during specimen transport such as sun exposure, heat, and shaking that could have altered the eggs in the case of Sadun and Buck [7], while further possible reasons might be attributed to species differences of *Paragonimus.* Korea is endemic mainly for *P. westermani* [25] whereas in Laos *P. heterotremus* is the main species [3], [17], [26], [27].

Our study is limited by its rather low sample size and thus differences between the diagnostic techniques might be underestimated. There might be paragonimiasis patients misclassified due to low or varying numbers of expelled eggs requiring examination of multiple samples or ectopic paragonimiasis [4], [7], [8] which might have diluted our results. We did not include feces samples in our investigation nor did we further investigate fluorescence microscopy, bleach concentration, and cold ZNS techniques. We did not investigate how time might affect *Paragonimus* eggs fixed in stored ZNS slides. We did not record the individual working time used per slide and therefore cannot give a variance. Since microscopy depends on examiner's experience it might initially take slightly longer when low-magnification ZNS microscopy is introduced.

Our cost effectiveness analysis did not account for capita costs and possible differences in quality-adjusted life years (QALYs) due to increased risk of TB transmission among laboratory technicians which both would further increase the cost-minimization by the ZNS. Another important differential diagnosis for TB is lung cancer which however remains challenging for resource-limited settings [28] and was not included since in Laos pathohistological diagnosis is limited to few central hospitals and unfortunately specific treatment is not yet available.

In conclusion, the current study, in contrast to previous reports, documents the usefulness and validity of the ZNS technique for detection of *Paragonimus* eggs. It appears to have superior sensitivity to the standard WF microscopic examination and has the best cost-effectiveness. Furthermore, ZNS examination for paragonimiasis does not carry biosafety risks and allows better post-test quality control. In addition to its use for the diagnosis of TB, we also recommend routine examination for *Paragonimus* eggs of each slide with the 10× lens (100× magnification) in geographic areas where paragonimiasis may be endemic. Its integration into the standard TB diagnostic procedure could help to reduce the misdiagnosis of sputum-negative TB due to paragonimiasis and could contribute to delineate endemic areas for this neglected parasitic infection.
